# Cross-Species Comparisons of Nanoparticle Interactions with Innate Immune Systems: A Methodological Review

**DOI:** 10.3390/nano11061528

**Published:** 2021-06-09

**Authors:** Benjamin J. Swartzwelter, Craig Mayall, Andi Alijagic, Francesco Barbero, Eleonora Ferrari, Szabolcs Hernadi, Sara Michelini, Natividad Isabel Navarro Pacheco, Alessandra Prinelli, Elmer Swart, Manon Auguste

**Affiliations:** 1 Institute of Biochemistry and Cell Biology, National Research Council, 80131 Napoli, Italy; swartzwe@colorado.edu; 2 Department of Biology, Biotechnical Faculty, University of Liubljana, 1000 Ljubljana, Slovenia; craig_mayall@hotmail.co.uk; 3 Institute for Biomedical Research and Innovation, National Research Council, 90146 Palermo, Italy; andialijagic@gmail.com; 4 Institut Català de Nanosciència i Nanotecnologia (ICN2), Bellaterra, 08193 Barcelona, Spain; fra.barbero@gmail.com; 5 Center for Plant Molecular Biology–ZMBP Eberhard-Karls University Tübingen, 72076 Tübingen, Germany; eleonora.ferrari2018@gmail.com; 6 School of Biosciences, Cardiff University, Cardiff CF10 3AX, UK; hernadi222@gmail.com; 7 Department of Biosciences, Paris-Lodron University Salzburg, 5020 Salzburg, Austria; sara.michelini@sbg.ac.at; 8 Institute of Microbiology of the Czech Academy of Sciences, 142 20 Prague, Czech Republic; natividad.pacheco@biomed.cas.cz; 9 AvantiCell Science, Ltd., Ayr KA6 5HW, UK; alessandra.prinelli@gmail.com; 10 UK Centre for Ecology and Hydrology, Wallingford OX10 8BB, UK; elmswa@ceh.ac.uk; 11 Department of Earth Environment and Life Sciences, University of Genova, 16126 Genova, Italy

**Keywords:** environmental models, human cells, innate immunity, markers, NPs testing

## Abstract

Many components of the innate immune system are evolutionarily conserved and shared across many living organisms, from plants and invertebrates to humans. Therefore, these shared features can allow the comparative study of potentially dangerous substances, such as engineered nanoparticles (NPs). However, differences of methodology and procedure between diverse species and models make comparison of innate immune responses to NPs between organisms difficult in many cases. To this aim, this review provides an overview of suitable methods and assays that can be used to measure NP immune interactions across species in a multidisciplinary approach. The first part of this review describes the main innate immune defense characteristics of the selected models that can be associated to NPs exposure. In the second part, the different modes of exposure to NPs across models (considering isolated cells or whole organisms) and the main endpoints measured are discussed. In this synergistic perspective, we provide an overview of the current state of important cross-disciplinary immunological models to study NP-immune interactions and identify future research needs. As such, this paper could be used as a methodological reference point for future nano-immunosafety studies.

## 1. General Introduction: The Need for Studying Nanoparticle–Immune System Interactions

Over the last twenty years, there has been a significant growth in the research, development, and production of engineered NPs [1]. When materials are downsized to the nanoscale, novel physical and chemical properties emerge, conferring them with new and unique behaviors. Depending on their nature (e.g., composition, size, shape, surface state), these materials have remarkable optical, magnetic, electrical, catalytic, structural, and chemical properties, which can be exploited in many different sectors such as automotive, agricultural, pharmaceutical, and biomedical fields [2,3,4,5]. It is estimated that the global nanomaterial production in 2014 was between 0.3 and 1.6 million tons, with SiO_2_, TiO_2_ and ZnO nanomaterials being the most abundantly produced [6].

**Figure 1:**
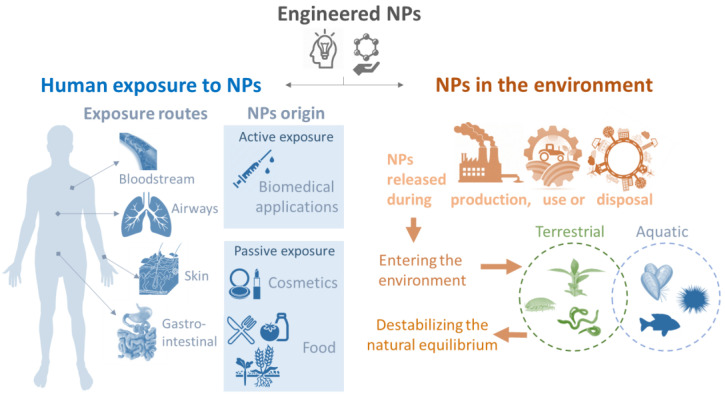
The different exposure pathways of engineered NPs that can interact with human or environmental species.

The wide utilization and increasing production of NPs has inevitably lead to an increase in humans and environmental exposure to these materials although exposure routes are not necessarily identical for different organisms. The expected increased exposure in human and environmental organisms has given rise to concerns regarding potential safety risks. The main exposure routes to NPs in both humans and environmental species are highlighted and summarized in Figure 1.

In humans, the first main exposure pathway is via intentional introduction of NPs, for instance during medical administration. The ability of some NPs to interact with molecular and cellular processes and to be target specific makes their use in drug delivery an attractive application. They have long been known to play an effective role in vaccination, acting not only as antigen carriers, but also as adjuvants that activate innate immunity and thereby increase the efficacy of antigen presentation [7]. They can also be valuable tools in medical imaging and diagnosis, and innovative new therapies [8]. Alongside the potential benefits of nanoparticle-based therapies, there is also a risk associated with parenteral introduction of novel substances, and thus there is a need to ensure that NPs will not negatively impact the normal functioning of the immune system [9,10,11,12,13]. Other interactions can arise from passive exposure such as through cosmetic products or food. Although NPs will likely first interact with epithelial and mucosal barriers, in some cases they are able to cross these barriers or potentially cause adverse effects, for example by interacting with the natural gut microbiome [14]. 

Although most NPs are not directly applied in the environment, many NPs used in consumer products or industry are expected to be released into the environment during production, use or during the disposal of products containing NPs [15]. Over the past decade, an increasing number of products containing NPs have been introduced into agricultural practices with the aim of increasing crop yield and reducing production costs [16]. In addition, the use of wastewater treatment plant biosolids as crop fertilizers can facilitate release of NPs into the terrestrial environment leading to exposure in soil organisms [17]. NPs can also reach aquatic environments, including seashores, through landfill leachates, or direct disposal of wastes (e.g., consumer products containing plastics) [18]. Once in the water, NPs can remain in suspension in the water column, interacting with planktonic organisms, or due to interactions with organic matter and/or their higher density, NPs can aggregate and deposit on the seafloor. This has been reflected by several models predicting NP concentrations within different regions which showed higher concentrations of NPs in sediments than surface water [19]. Therefore, benthic and sediment dwelling organisms are expected to be exposed to NPs, due to their feeding habit (e.g., filter, deposit feeders) [20]. In addition, some marine invertebrates possess an open (or semiopen) circulatory system, which is in direct contact with the external environment, eventually contributing to increased exposure. 

Considering the many possible exposure and entry routes of NPs, defining common parameters for assessing organism-NP interactions is fundamental for allowing comparisons at different taxonomic levels. Innate immunity is a shared feature for every multicellular organism and the effector mechanisms of the innate immune system are the first line of defense that detect and protect the body from nonself objects such as NPs [21,22,23]. As is the case for natural pathogens, NPs have the potential to induce an immune response. In cases where NPs can elicit an immune response, there is a need to study the type and degree of this response, and the NP-immune interaction mechanisms rather than remaining limited to only measurements of acute toxicity. Comparative immunology, by its multidisciplinary approach may unravel fundamental mechanisms activated by NPs and help further global understanding regarding the effects of NPs. 

**Figure 2:**
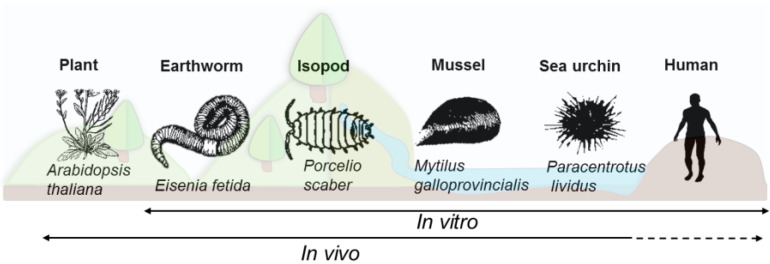
The different models discussed in the current review, and their main experimental usage in the laboratory: in vivo (whole organism experiments) and in vitro (isolated cells or cell lines).

Experiments in a laboratory are first necessary to allow the understanding of basic mechanisms under controlled conditions. However, carefully chosen models and assessment parameters are important with regard to future translocation to more realistic environmental exposure. To this end, models within this review have been selected which can be good indicators and representatives of their regional and global distribution and which are easy to maintain under laboratory conditions. Environmental models can be therefore compared across taxa and even to human cells, through both in vitro and/or in vivo approaches according to the model possibilities (Figure 2). Plant models are a compelling place to begin for assessment of NP-immune interactions. In particular *Arabidopsis thaliana*, a small flowering plant belonging to the Brassicaceae family, which is widely used in crop science studies and was also the first plant genome to be fully sequenced [24,25,26]. Among terrestrial invertebrates, earthworms belonging to the family Lumbricidae *(Eisenia fetida)* are abundant in the soil and play an essential role in soil formation, by facilitating nutrient cycling, fragmenting biomass and aeration of soil through bioturbation [27,28]. Similarly, terrestrial isopods, such as *Porcelio scaber*, are crustaceans which evolved to live on land, inhabiting the top-soil level. They are decomposers and play an important role in returning nutrients to the soil [29,30,31]. Their feeding habits makes it likely they will come into contact with environmental pollutants, including NPs, and thus represent interesting model species to study these interactions. The Mediterranean mussel *Mytilus galloprovincialis* and the sea urchin *Paracentrotus lividus* are both sessile marine invertebrates. Mussels are able to filter large quantities of water which they use for breathing and feeding, while sea urchins graze on the seafloor layer. These qualities, as well as the ease with which they can be harvested along seashores, make these good models in which to study invertebrate interactions with NPs [20,32,33,34].

This work is supported by the EU PANDORA project [35], which devoted effort to study the effects and mechanisms of action of NPs on the innate immunity of different models from across the tree of life. The general outcomes of the project were previously reported, summarizing the main findings but also to set future perspective and research direction in this field [21,36]. The remainder of this review will focus on the translatable aspects of experimental methodology, parameters and endpoints used, the suitably of the selected models when considering investigating NP effects, and the possibilities regarding research at the whole organism level (in vivo) or with isolated cells (in vitro). 

Here we aim to: (i) give a short overview of the characteristics of various relevant innate immune models from across tree of life; and (ii) provide a comparative analysis of the methods used to study the interaction of NPs with these innate immune models.

## 2. Short Description of the Innate Immune System for the Models of Interest

### 2.1. Generalities and Conserved Innate Immune Traits across the Selected Models

The ability to mount an immune response against external threats is a characteristic of every living organism. While increasing levels of immune complexity are found in higher organisms, at every stage of evolution there is present a basic initial host defense that has been characterized as innate immunity. Innate immunity is a fast, standardized, nonspecific response which includes multiple levels of defense mechanisms, beginning with physicochemical barriers (e.g., shell, mucosal or epithelial barrier) [37,38]. Further mechanisms of defense rely on dual components of the immune system, the immune cells (e.g., monocytes, macrophages for vertebrates, or hemocytes, coelomocytes for invertebrates) and the production of humoral factors. Innate immune cells found in the circulating fluids of invertebrates can have different names and the cellular portion of their innate immunity relies on these unique cells. These cells can be subdivided into different cell populations, such as granular or hyaline cells, and they have distinct roles and can trigger a specific response upon encountering threatening nonself material. Only plants lack these specialized immune cells, but in plants all the cells are believed to be able to mount a defensive response to foreign attack [39]. Complex machinery, including cells and humoral factors is involved in recognition of nonself material, and especially in detecting domains called pathogen/microbe associated molecular patterns (PAMPs/MAMPs) that are typically displayed on the surface of bacterial, fungal, and parasitic organisms and virus-infected cells. Host recognition of nonself will involve a large range of cell membrane bound and scattered pattern recognition receptors (PRRs). Although the distinctive PRRs can vary between models, the main concept is consistent, and different PRRs share a similar role upon recognition of nonself particles. PRRs in humans, much like their invertebrate homologues, are responsible for initiating innate immune cell responses including the initiation of phagocytosis or endocytosis, cellular motility, and beginning the processes leading to inflammatory reactions [40]. Upon successful recognition, the pathogen detecting cell will initiate a process of destruction or sequestration to eliminate eventual danger, and later repair the stress or damage caused by this unexpected material. Most invertebrate immune cells, similarly to human macrophages or monocytes, are involved in phagocytosis, which remains one of the most efficient mechanisms to clear nonself material. The induction of some global defense mechanisms can be easily observed across different models, such as the production of reactive oxygen species (ROS) and nitrogen radicals (RNS), synthesis and secretion of antibacterial and antifungal proteins, cytokine-like proteins, and hydrolytic enzymes. Antimicrobial peptides (AMPs—small cationic, amphipathic molecules) are very well studied and highly involved in invertebrate immunity. They can tag objects or induce direct destruction by destabilizing biological membranes, which make them effective against large range of unicellular organisms like bacteria, yeast, fungi, and also some protozoans and enveloped viruses [41]. Circulating fluid also contains a large panel of enzymes (released by immune cells) with hemolytic, proteolytic and cytotoxic roles (one of the most common being lysozyme).

The encapsulation of foreign objects and activation of enzymatic cascades that regulate melanization and coagulation of hemolymph are also common defense mechanisms encountered in invertebrates. Indeed, phenoloxidase is considered among the most important components of the invertebrate immune system, especially in insects and crustaceans. The phenoloxidase cascade produces the antimicrobial molecule melanin, as well as inducing multiple potent bioactive agents such as peroxinectin and ROS, that aid in phagocytosis and cell adhesion. Melanization is essential in wound healing, encapsulation, and nodulation. Proper modulation of this enzyme is crucial to ensure survival of the organism. The majority of species activate the phenoloxidase cascade using the proPO enzyme [42,43,44].

Although general immune features are conserved across the previously described models and organisms, adaptations exist in each case that address the organism particular vulnerabilities and environmental stresses. These adaptations occur according to the organism’s lifestyle, habits and need, which might cause certain parameters to be more important than others in some species to deal with threats some models are more likely to encounter. In line with this, the next section aims to report the main mechanisms and characteristics of innate immune responses for the selected models, and in particular those known to be activated upon exposure to NPs. A summary is presented in Table 1.

### 2.2. Model Specific Immune System Characteristics

#### 2.2.1. Plants

Plants lack specialized mobile immune cells, and every cell is believed to be capable of initiating an immune defense against pathogens and invaders. Two layers of innate immune responses, i.e., pattern triggered immunity (PTI) and effector-triggered immunity (ETI), provide an efficient defense that keeps most pathogens and external attacks under control [39,45]. The activation of PTI relies on PRRs perceptions of MAMPs/PAMPs to trigger complex immune responses. PRRs are solely on the cell surface of plant cells and among them the most studied is the plant flagellin receptor-FLS2 [46,47,48,49,50,51]. The second line of defense, ETI, is mediated by nucleotide-binding domain leucine-rich repeat (NB-LRR) disease resistance proteins (NLR), which induce defense responses leading to a hypersensitive programmed cell death [52]. NLRs can detect effectors directly or by indirect surveillance of the effector action on other host target proteins [39]. Both lines of defense share parts of their defense signaling pathways [53,54]. After pathogen detection, multiple morphological and physiological responses are induced, such as ion fluxes over the plasma membrane, including Ca^2+^- and H^+^-influx; production of ROS and antimicrobial compounds (phytoalexins); activation of mitogen-activated protein kinases (MAPKs) and calcium-dependent protein kinases (CDPKs). In consequence, the transcriptome will be reprogramed by activation of a subset of transcription factors; callose deposition; stomatal closure; restriction of nutrient transfer from the cytosol to the apoplast and programmed cell death [55,56]. Phytohormones such as salicylic acid, abscisic acid, jasmonic acid, and ethylene have a critical role in the plant’s responses to specific pathogens [57]. Defense hormones can be transported within and between plants to alert distant tissues and confer systemic immunity [55,58].

#### 2.2.2. Earthworms

Earthworms are protostome animals that have large coelomic cavities throughout the length of the animal. The coelomic cavity is typically nonsterile, open to the outer environment through dorsal pores, allowing the entrance of fungi, bacteria, and protozoans. Coelomocytes can be classified into two major cell types: amoebocytes and eleocytes. Amoebocytes (hyaline and granular) are involved in various immune responses including phagocytosis, encapsulation, and the production of antimicrobial molecules [59,60]. Eleocytes display more nutritive and accessory functions [59,61]. Three types of PRRs have so far been identified: coelomic cytolytic factor (CCF) [62], toll-like receptors (TLR) [63], and lipopolysaccharide-binding protein/bacterial permeability-increasing protein (LBP/BPI) [64]. CCF has two recognition domains that can interact with bacterial or fungal MAMPs, which in turn triggers the proPO cascade [42,65]. A range of antimicrobial molecules including lysozyme and the hemolytic proteins fetidins and lysenins are involved in the elimination of the microorganisms [66,67,68,69].

#### 2.2.3. Isopods

The hemocytes of *Porcellio scaber* originate in the hematopoietic glands located along the animals dorsal vessel, and can be split into granular and hyaline hemocytes [70,71]. Hyaline (absence of granules) hemocytes are mainly responsible for phagocytosis [71]. Semigranular cells also show some phagocytic ability but seem more involved in encapsulation and nodulation. Granular cells are predominantly involved with the phenoloxidase system [72], and along with semigranular cells are thought to produce AMPs and be involved in antioxidant defense [73,74]. In *P. scaber*, the PO cascade is initiated by hemocyanin [44]. In addition, for defense they are able to produce, RNS, ROS, and AMPs like other invertebrates [75,76]. Genomic mining of the terrestrial isopod, *Armadillidium vulgare*, revealed genes for specific AMPs including anti-lipopolysaccharide factor (ALF) 1 and 2, crustin 1, 2, and 3, and I type lysozyme, and pathogen recognition genes C-type lectins 1, 2, and 3 and peroxinectin-like A and B [77]. 

#### 2.2.4. Mussels

The innate effector cells of *Mytilus*, hemocytes, are composed of granulocytes and hyalinocytes. Mature granulocytes are among the first lines of cell defense for the elimination of invaders via phagocytic processes [78]. In *Mytilus*, a large range of PRRs, anchored on the cell outer membrane and secreted are encountered, with lectins the most dominant group. Other classes of soluble PRRs are found, like C-terminal fibrinogen related domain-FReD-containing proteins, which have been shown to improve the rate of phagocytosis. TLRs and peptidoglycan recognition proteins-PGRPs, and others have been recently discovered but further study remains to be done to properly appraise their mechanisms of action (see [79] for more details). Moreover, *Mytilus* possesses the complement system pathway and relies on the involvement of C1qDC (C1q domain-containing) proteins [80]. Several signaling transduction pathways have been reported to be present in bivalves such as the mitogen-activated protein kinase (MAPK), nuclear factor-κB (NF-κB), the complement component, the toll pathways, and the JAK-STAT pathway (reviewed in [79]). Additionally, as with other species, hemocytes can trigger the production and release of several factors such as ROS, nitric oxide-NO, hydrolytic enzymes (e.g., lysozyme), and AMPs. Several AMPs have been identified in *Mytilus* such as mytilin, myticin, mytimicin (with an antifungal and/or antibacterial role) and defensins [81]. In extreme cases and for larger objects, hemocytes can encapsulate foreign matter via the coordination of several hemocytes and the release of cytotoxic products (enzymes or ROS) to degrade the material, followed by cellular reabsorption of the debris [82]. Finally, the proPO cascade, while present, remains relatively unstudied in bivalves [83]. 

#### 2.2.5. Sea Urchins 

Sea urchins contain circulating immune cells called coelomocytes which can be subdivided into four classes and are able to infiltrate into different tissues [84,85]. The macrophage-like phagocytes can encapsulate and internalize nonself particles, red amoebocytes release the bactericidal pigment echinochrome A, and white/colorless amoebocytes operate the cytotoxic/cytolytic response, while vibratile cells most probably degranulate and trigger immune cell aggregation [85]. Recently, their genome sequences have revealed the presence of a vast array of immune-related genes, including those coding for PRRs such as TLRs, NLRs or SRCR domain-containing proteins, and complement proteins (Complement C3 homologue) [86,87]. Moreover, lectins are also important in sea urchins and in addition to their role in opsonization, they show lytic functions, and are involved in wound repair [88]. Sea urchins contain several humoral factors including hemolysin and agglutinin which can be induced upon cell activation. ROS are also produced during immune responses. Echinoderms possess many different AMPs with various modes of action depending on the species, among them paracentrin 1 in *P. lividus*, showing an antimicrobial role [89]. Interestingly, the phagocytes can contain AMPs but they are not released into the extracellular medium, instead they play a role within the phagolysosome [88]. Finally, sea urchins belong to deuterostome lineage which makes them phylogenetically near to chordates, sharing several common traits with mammalians, especially with regard to cytokine production [90,91]. 

#### 2.2.6. Human Cells

Human and other mammalian immune responses are organized within two branches: the innate immune response which is characteristic of all eukaryotes, and additionally a highly specific adaptive immune response, individualized for distinct pathogens. As NPs do not display highly specified and unique surface patterns, it is the innate branch of human immunity that is tasked with responses to NP exposure. Nonself particles and pathogenic threats that enter human circulation may activate humoral components such as AMPs and complement alongside innate immune cells. Cells participating in the human innate immune response include granulocytes, such as neutrophils (which primarily function to overwhelm pathogenic invaders through large numbers and phagocytic mechanisms), and myeloid-derived cells, including monocytes, macrophages, and dendritic cells (DCs). Monocytes represent about 2–8% of the leukocytes in circulation at any given time [92] and generally patrol the circulatory system for signs of foreign particles or internal damage. They can be recruited in tissue via resident cells releasing chemokines such as CCL2 [93]. As monocytes attempt to engulf foreign particles by phagocytosis, simultaneous chemokines and cytokines are secreted that signal for a broader inflammatory and immune response. They can be further involved in the resolution of an inflammatory reaction, assisting in tissue repair [94]. Macrophages, are tissue resident and represent up to 15% of the cells in a given tissue [95]. Functionally two broad classes of macrophage exist, M1 which display a more inflammatory phenotype involved in early immune response (killing and defending); and later M2, which display more phagocytic and tissue repair oriented traits [96,97,98]. Finally, DCs are known as antigen-presenting cells, acting as the bridge between human innate and adaptive immunity. They play a role in the generation of pathogen-specific T-cells and B-cell antibodies. Of the PRRs in humans, toll-like receptors (TLRs) play the most prominent role in the detection of extracellular pathogens [99], where they recognize substances such as bacterially associated carbohydrate patterns or RNA sequences associated with viruses [100]. Other PRRs that can be found on the membrane of human innate immune cells include scavenger receptors, which detect various polymers and lipoproteins [101], and C-type lectin receptors including dectin-1, which recognizes B-glucan components of various fungi [102]. In addition, they are some intracellular PRRs found in cytosol, such as NOD-like receptors (NLRs) and rig-I-like receptors (RLRs), which recognize a large range of PAMPs [103,104,105]. The most notable difference with invertebrates, is the diversity and number of types of cells involved in the immune response. 

## 3. Parameters Assessed: From NPs to Innate Immune Responses

### 3.1. NPs: What to Consider When You Use a Biological System?

**Figure 3:**
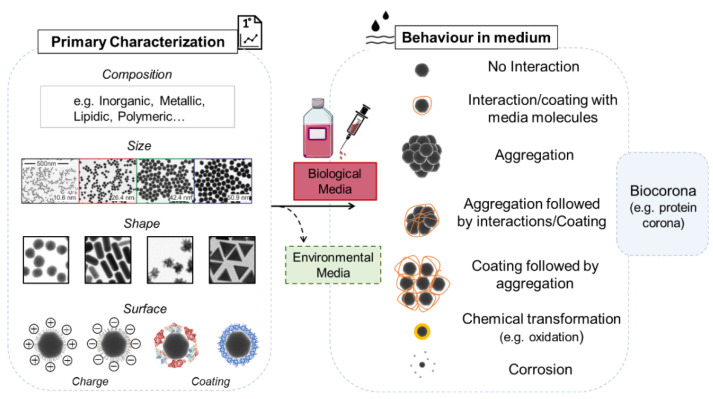
The different characteristics of NPs and parameters to investigate when they are in suspension media for laboratory experiments.

The physico-chemical characteristics of a NP and its behavior in different exposure media are fundamental considerations when attempting to understand the interactions of NPs within a biological model. It is important to take into account that the relatively large surface area to volume, the low coordination of atoms at the surface, and their colloidal nature cause NPs to display physical and chemical characteristics that differ from their bulk counterparts. It is also fundamental to understand the characteristics of the final object that living organisms will encounter and to correlate the pristine and final NP features with the potential effects on living organisms. The main NP characteristics to be considered are reported in Figure 3.

#### 3.1.1. Primary Characterization

The first determination of primary characteristics includes the description of the material composition, the nominal size, shape, and surface charge (zeta potential). Moreover, characterization of NP coating and other surface modifications are crucial to consider (Figure 3 left panel). 

#### 3.1.2. Behavior in Medium

In addition to the known properties of a chosen NP following synthesis, once exposed to biological conditions (e.g., medium or circulating fluid), NPs can display unpredicted new characteristics. NPs can have a propensity to move towards a more stable thermodynamic state via different means: aggregation (which can mean escaping from the nanoscale), formation of a coating composed of various molecules, chemical transformations, particle corrosion, and dissolution [1]. All these transformations can change the identity of the NP or produce new chemical entities (e.g., reactive metal ions), modifying their behavior and their potential associated risk and interactions (Figure 3 right panel). Therefore, the determination of NP characteristics in exposure medium needs to be assessed. This generally includes the aggregation state (Z-average), the change in surface charge (zeta potential), and the dispersion index (PdI). Moreover, the evolution over time of these parameters can also be of value for a full appraisal of the NPs dynamic in the exposure medium. All these analyses are usually performed using DLS (dynamic light scattering) analysis, or electron microscopy (TEM and SEM) depending on the material being investigated. Additionally, careful controls have to be performed in order to avoid artifacts due to the presence of chemicals, often used to stabilize the particles [106,107], or contaminants, such as bacterial lipopolysaccharide (LPS), that can cause false positives in an immune assay [108]. 

Another routinely measured parameter is the presence and composition of the molecular biocorona, where components of biological fluids can be adsorbed by the NP, forming a corona on its surface. Usually, they are believed to be mostly constituted by proteins (protein corona -PC) but other macromolecules including lipids present in the medium can also contribute to its formation. The presence of this supplementary layer on top of the NPs can in turn affect the NPs behavior and interactions with the surrounding media. However, this corona depends on both the biological fluid (plasma, or otherwise) composition and the properties of the NPs, including size, curvature, surface functionalization, and charge. The composition of the corona is theoretically divided into the soft corona (weakly bound) and the hard corona (tightly bound), but it is dynamic and the ligand on the top can be exchanged and replaced over time, according to the affinity of the macromolecule for the NPs [109,110]. The PC is the biological identity of the NP and represents what cells “see” and with which they will interact [111,112,113,114,115,116,117,118]. Consequently, recognition by immune cells can be different and specific from one type of NP to another, which means that they will interact with the protein on the surface rather than the NP itself. This results in the triggering of defense mechanisms different from those observed in medium free of proteins. This does not apply only to mammalian plasma, but it has been demonstrated in the biological fluids of different terrestrial and marine invertebrates, including earthworms, bivalve, and sea urchins, in which the composition and effects on immune parameters appeared different for each NP type [119,120,121,122]. For these reasons, the PC is an important parameter to consider under laboratory conditions and needs to be characterized with precision during the exposure event. 

All the previously cited characteristics can also be applied to environmental media [123,124,125,126]. The NPs will be subjected to other factors like abiotic physico-chemical parameters (such as pH, ionic strength, temperature) which can influence their dispersion, aggregation, agglomeration [127,128], interaction with molecules present in their environment, and adsorption to macro-organic matter (e.g., eco-corona) [117]. Scientific literature is being produced on the physico-chemical transformation of NPs due to their exposure to aquatic and terrestrial scenarios, correlating the environments and particle properties with the observed changes. Consideration of this should be taken into account in future studies working with environmental scenario experiments [129,130,131,132]. 

### 3.2. Models, Cell Culture and Mode of Exposure

**Figure 4:**
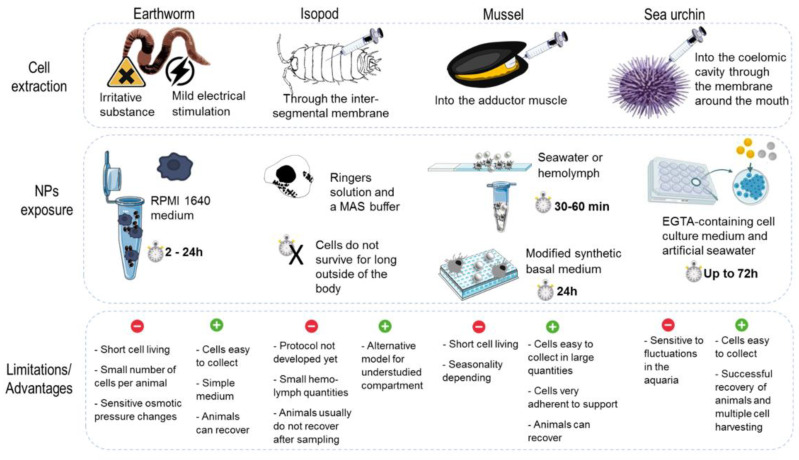
The different in vitro approaches and NPs exposure parameters encountered across the selected models.

**Figure 5:**
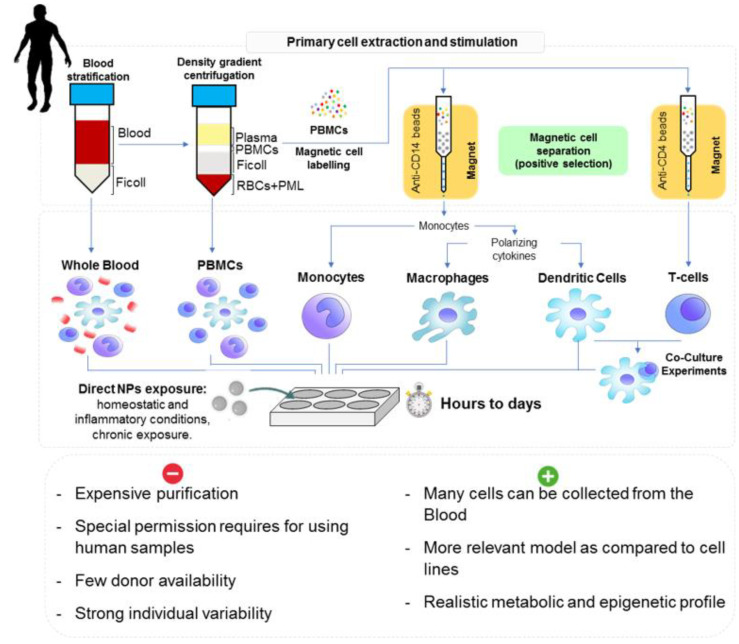
The different in vitro NP exposure possibilities available for human primary cells and the procedure of cell extraction and preparation (Reprinted with permission from Michelini et al. (2021) [153]. Copyright 2021, Copyright Royal Society of Chemistry).

In experimental science, the use of in vitro assays is being promoted as sustainable alternative for a large range of product testing, including NPs, following the 3R principle (replacement, reduction, and refinement). The extraction of immune cells, separation, culture and feasibility to maintain such isolated primary cells varies across models. To illustrate, a summary of the different methods of cell harvesting and exposure for both invertebrates and human primary cells are reported in Figure 4 and Figure 5. 

#### 3.2.1. Nonmammalian In Vitro Assays

In many invertebrates, coelomocytes/hemocytes act as the first line of defense against nonself objects. The induction of functional responses with these cells is often rapidly observed, helping to counteract the limitation of the relative short-term lifespan of cells in cultures (ranging from a few hours to a few days depending on the model). As a natural defense mechanism, earthworms can extrude their coelomic fluid, through dorsal pores. Therefore, coelomocytes can be extracted by mild electrical stimulation or by exposing the animals to an irritative substance [133,134]. Immediately upon collection, the coelomocytes need to be stabilized in a culture medium in order preserve cell viability. Recent studies show that RPMI 1640 medium is the optimal medium for earthworm coelomocytes culturing, as well as the assessment of NP toxicity towards coelomocytes [135,136]. Critical for the successful culturing of coelomocytes is the adjustment of osmolality of the medium so that it reflects that of the coelomic fluid [137,138,139]. Exposure time for in vitro assays depends on cell viability and may range from between 2 to 72 h, with 24 h being the optimum time for cell cultures in RPMI 1640, according to some investigations [136,137,140]. For terrestrial isopods, the culturing of hemocytes did not show hopeful results yet. Hemolymph can be collected by puncturing through the intersegmental membrane on the dorsal side of the isopod with a sterile needle and collecting the hemolymph with a micropipette. With the use of ringers solution and a MAS (mitochondrial assay solution) buffer, cells appeared to hardly survive for even a few hours outside the body. The selection of a suitable medium is still needed to be identified and adapted for keeping hemocytes alive without showing excessive levels of stress [141]. 

In marine invertebrates, in vitro experiments are much more abundant, in particular, experiments using hemocytes of the marine mussel *M. galloprovincialis* extracted via a non-invasive method. This method can provide a first line of investigation for testing several types of substances, including NPs [142,143,144,145]. *Mytilus* hemolymph is easy to collect via the adductor muscle and fluid quantities are sufficient (depending on the season, volume can be as high as several ml per animal) to perform various experiments [146]. Short-term exposures (≤1 h) have shown rapid activation of hemocyte functional parameters but longer exposure times (up to 24 h) have shown the induction of further immune or stress parameters. For short experiments, hemocytes can be maintained in a natural hemolymph or seawater suspension, in tubes or as monolayers on glass slides. Longer culture times were more successful when modified synthetic basal medium (Basal Medium Eagle) was used in microwell plates. These in vitro experiments are possible due to the cells ability to quickly adhere to supports (<20 min) [147,148]. Ex-vivo tissue explant has also been used (e.g., gills) to study the first interactions and potential uptake of NPs [149]. 

The coelomocytes from sea urchin once extracted are placed in cell culture plates and kept in EGTA-containing cell culture medium and artificial seawater [84,85,150]. The coelomocytes can be kept for a long period of time (over two weeks), with regular medium replenishment and without the addition of the special growth factors or nutrients [90,151]. 

Finally, as plants do not possess specialized mobile immune cells, in vitro research is not typically suitable/realizable, and the main experiments in laboratories are made on the full plant or tissues excisions (see next section). Each piece of tissue should respond upon exposure as each single cell is able to launch an effective immune response [152].

#### 3.2.2. Human Cell Models

The study of human cells offers a wide range of possibilities not currently developed for invertebrate models. Multiple cell types, coculture conditions, and cell maturation or differentiation programs exist to define more precisely the interactions with NPs. Several important models used in mammalian systems to test NP–immune system interactions are listed below. 

In vitro modelling of human innate immunity is usually conducted using monocytes, macrophages, or dendritic cells, as these cells are responsible for directing the innate immune response from pathogen recognition to phagocytosis to inflammation, and even to eventual antigen presentation and induction of adaptive immunity. Cell lines for each of these cell types exist and are frequently used due to their easy experimental repeatability and scalability, with THP-1 (monocytes) and U937 (macrophages) being the most frequently reported [154]. However, cell lines are truly limited to the representative phenotype observed at the time of culture, and even this is susceptible to mutations that do not represent the true reactivity of healthy human cells. More robust models of innate immune responsiveness utilize primary cells, which are collected directly from donors and may be isolated using techniques that select for the desired cell type. Primary cells are representative of an individual’s current in vivo condition, and lack the altered metabolic and epigenetic profile inherent to cell lines. Furthermore, as monocytes are found abundantly in circulation, and since they can be precursors for both macrophages and DCs, the differentiation of monocytes in culture into primary differentiated macrophages or DCs is an effective tool to create models of innate immune responses in vitro. However, models utilizing primary cells must contend with individual variability as the immune experience and capacity is different between donors [155]. 

The whole blood assay is one of the most simple and rapid tests for assessing the immune activating capacity of novel substances within a human system. Typically, blood is drawn from healthy donors and immediately exposed to the substance under investigation, with 250 µL of blood typically diluted in 750 µL of RPMI plus the tested material and incubated for 24–48 h [156]. Peripheral blood mononuclear cell (PBMCs) can also be isolated from the whole blood using Ficoll–Paque density gradient centrifugation [157]. Magnetic cell separation using some CD-4 beads can be used to isolate monocytes, and later growth factors can be added to differentiate macrophages such as macrophage colony stimulating factor (M-CSF) or DC GM-CSF and IL-4 [94,158]. 

The monocyte activation test (MAT) models (or using macrophage and DCs) can assess the exposure and response of monocytes to NPs, and many parameters may be assessed following activation [159,160,161]. Usually cells (in the range of ~500,000 cells/mL) in plate culture can be directly exposed to NPs added to the wells, and tests on monocyte/macrophage/DC activation are typically completed within 24 h. Oftentimes, PBMC culture is conducted in round bottom wells, which simulate a lymph node in which communication between myeloid cells and lymphocytes occurs. 

Finally, NPs can also interact with other cells present in blood, such as DCs, which link with adaptive immunity. Similar principles of the MAT test can also be applied for testing NPs, but also cocultures with T-cells of self or foreign origin [153]. These types of test can mimic autoimmunity or the mixed lymphocytes reaction and are of interest for the use of NPs in vaccines and immunotherapy [162]. 

Experiments considering primary isolated cells offer other advantages by representing a simplified model, limiting interfering factors, which could help to spotlight NPs mechanisms of action before performing further experiments; e.g., coculture, tissue models, or even using whole-organism in vivo experiments. Moreover, these in vitro experimentations allow easier comparison between models, particularly, with human cells. In addition to the commonly known pros for in vitro assessment such as cheap cost, fewer animals used and relatively fast results, a list of the more important pros and cons for each model, with special input for in vitro assays, is presented in Figure 4 and Figure 5, lower panel. Although they provide a simplified set up and can be used to try and understand some of the basic mechanisms, they do not represent the true exposure pathway. Additionally, large variations in the exposure time and the culture methods between different models persist. In invertebrates, the immune cells are usually easy to collect, except for isopods, and in large quantity. There are some species-specific difficulties in experimentation, such as molt cycles in isopods or seasonality with reproductive period in mussels that can impact immune measurements. Moreover, some cells are more sensitive and fragile to handle compared to others. Usually, the immune cells from invertebrates are viable for shorter times in culture, as basal parameters are quickly impacted. For humans, in addition to regulatory hurdles, donor availability is restricted for the obtaining of primary immune cells. Each donor is usually considered independently, which can reflect stronger variabilities in responses. However, from one whole blood sample many cells can be collected and offer a large range of possible assays after purification. 

### 3.3. Whole Model Exposure Experiments

**Figure 6:**
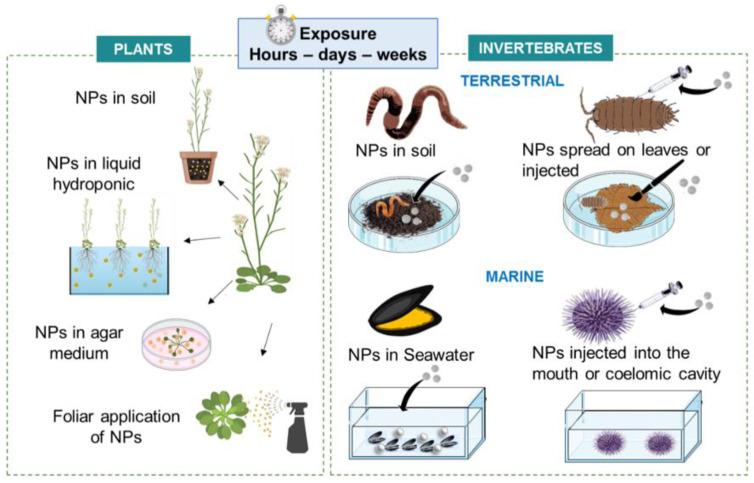
NP exposure approaches using the whole organism with the different exposure pathways across the selected models.

In vivo experiments allow for evaluation of the effects and mechanisms of action of NPs in organisms at different levels of biological organization (molecular, cellular, tissue level). They provide a realistic scenario of the exposure pathways as encountered under natural conditions. For controlled laboratory experiments, the mode of exposure to NPs needs to be adapted for each model; a summary is presented in Figure 6. 

These tests are usually conducted in environmentally relevant mediums (soil or water), through feeding experiments or through breathing and filtering experiments for aquatic species. As the selected models are usually easy to maintain in laboratory conditions for long periods of time, requiring little space and maintenance (e.g., feeding), the exposure time (acute, semichronic and chronic exposure) can range from hours, to days and weeks. 

Plants can germinate and grow directly in the presence of the NP in the growth medium, i.e., soil, hydroponic nutrient solutions or agar-solidified agents, or they can be exposed at subsequent development stages. Despite the presence of cell walls, that can represent a barrier preventing NPs entering into the plant cell and cytoplasm, NPs might be absorbed through root or leaf and be potentially transported to the shoot or to other points through the phloem (vascular system) [163,164,165,166]. NPs can be also dispensed onto the plants surface by foliar spray application [167]. After entrance into the leaf tissue, NPs can diffuse into the intercellular space, the apoplast, or membranes and cause secondary effects. Moreover, temporality is important, and it is necessary to understand the course of plant growth and development, from seed germination to root elongation and shoot emergence, in relation to NP exposure [168]. The following investigations can assess the NP uptake by cells and further nanophytotoxicity, focusing on the toxicity symptoms of plants. 

In vivo earthworm exposures are typically conducted in soils following well-described and standardized procedures (e.g., [27]) that can also be applied for NPs [140,169]. However, care must be taken when it comes to the mixing of NPs with soils, adjusting the parameters depending on the form (i.e., as solution dispersion or as powder) in which the NPs are supplied [170]. Furthermore, coexposures with infectious microbes are also important in order to establish whether an exposure to NPs has an effect on the ability of a host to maintain immunity [171]. A methodological approach to investigate the impact NPs have on the earthworm’s ability to maintain immunity when coexposed with infectious bacteria has been recently established [140]. 

A major benefit of working with the terrestrial isopod, *P. scaber* is that they are able to be exposed to the NPs in a manner similar to how they would be exposed in nature. NP suspensions can be spread on leaves that *P. scaber* eat, and both the leaf and animal are then placed in a petri dish. During the experiment, feeding rate, defecation rate, and mortality can be monitored. This also allows for modelling of real-world impacts of NPs on the organism from behavioral changes, like feeding avoidance and mortality, to cellular immune responses. However, the gut of *P. scaber* is covered in a thick cuticle which is believed to stop the translocation of NPs from the gut into the hemocoel where the hemocytes are, so the immunological effects of the NPs might not be seen when ingested [172]. There is the possibility for an alternative exposure scenario, with injection experiments delivering substances directly into the animals hemocoel, allowing for the study of the direct interaction of a known concentration of NPs with the hemocytes. This ensures NP and immune cell interaction [141,173]. 

Mussels are suspension feeders and are able to filter large amounts of water (up to 3 L per hour) implying that, in a short period of time, they can easily uptake the NPs present in the seawater of experiment tanks. For this reason, the NPs can be directly added to the seawater and the ventilation system allows for constant movement of water within the tank. To study the first immune defense response, short term experiments (24 h to 96 h), have been shown to be sufficient to induce the activation of the immune system [142]. Moreover, the use of artificial seawater (ASW) implies the absence of organic matter or other substances that could interact with the NP suspension; together with a constant salt content and as such are reproducible for all periods of the year. The experiments are mainly conducted in the spring and summer periods where mussels are at their healthiest. During experiments, mussels are not fed and can readily survive several days without feeding [146]. This is necessary for NP experiments, as the presence of microalgae could interact with the NPs. In this context, the study relies only on the uptake of the NPs in seawater. To mimic a more realistic exposure, other studies have performed longer-period experiments to consider the interactions between food intake and the NPs, but this generally focused more on physiology and tissue changes and not strictly the immune response [148,174]. Biological uptake routes are dependent on NP properties and may occur as direct uptake in gill tissues and/or through transference from the cilia to the digestive system. Moreover, the agglomeration of particles in seawater has been shown to facilitate NP ingestion by suspension feeding bivalves, and their potential translocation from the gut to the circulatory system [175,176]. However, this internalization pathway seems to vary according to the NPs and some can be captured and excreted in pseudofaeces (mixture of mucus and undigested particles) before arriving to the stomach, resulting in lower tissue accumulation and higher depuration [177].

The existing in vivo studies utilizing sea urchins mainly focus on the immune status of the animal after exposure to NPs via the injection of the NP suspension into the mouth or directly into the coelomic cavity (through the soft peristomal membrane surrounding the mouth). Consequently, NPs injected orally partially cross the intestinal epithelium, invade the coelomic fluid and are then engulfed by phagocytes, while the remaining particles pass the digestive system and can be excreted [178]. On the other hand, NPs injected into the coelomic cavity can directly interact and be recognized by phagocytes [85,179].

In general, the in vivo passive exposure experiments consider more realistic exposure pathways (feeding, breathing) of NPs. However, for more simplistic set up and to be sure that NPs encounter immune cells, NP suspensions can be also injected into the animal. Results obtained from in vivo tests can provide a good proxy of interactions of immune cells in situ and thereby in vivo tests are crucial to resolve the issue of whether NPs pose an immune threat to living organisms. These models have been shown to be easy to maintain in the laboratory, and exposure experiments allow for the effects of NPs to be studied at different levels of the organism. For future experiments, mesocosms will help to mimic environmental scenarios before further studies in the environment.

### 3.4. Innate Immune Parameters of Interest 

**Figure 7:**
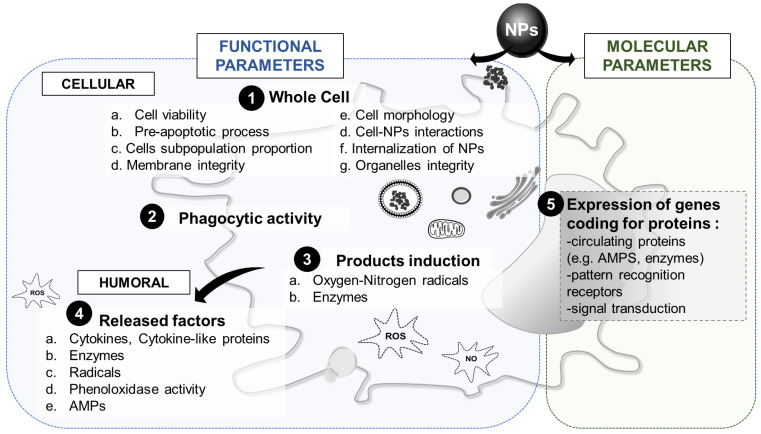
Summary of the different endpoints measured in immune cells after exposure to NPs.

As highlighted in Figure 7, a variety of endpoints can be used to compare immune system–nanoparticle interactions between different models. This includes functional responses, which comprise the biochemical assessment of cellular and humoral responses, and molecular responses that aim to evaluate changes in the expression of immune-related genes. Because there are many methods available to quantify functional responses, here we provide a comparative overview of these methods to show which are most appropriate for the purpose of NP testing and cross-species comparisons (see Table 2).

#### 3.4.1. Whole Cell Response

Parameters looking at the whole immune cell concerns the immune cells viability, the membrane integrity, all the different types of interactions they can have with NPs, and their potential changes in morphology (Figure 7, first point). The first important cellular responses that can be used to investigate nanoparticle—immune system interactions is (immune) cell viability. Although cell death and apoptosis are part of a normal immune response, studies have shown that NPs are able to cause excessive mortality in immune cells with possible adverse effects on immunocompetence. There are several methods available that can measure cell viability (Table 2). A common method used in human cell lines is the measurement of the release of LDH or ATP through biochemical assays [153,182]. An alternative is the staining of living or dead cells using fluorescent probes (e.g., fluorescein diacetate–FDA or propidium iodide–PI) for observation using flow cytometry fluorescent microscopy. In some models, cell viability may also be studied by measuring metabolic activity through cell-permeable fluorescent reduction such as CTB or the colorimetric MTT. In some models the use of counter stain dye such as trypan blue or nigrosine [141,192] or the use of DNA-binding florescent dyes are alternative methods for assessing cell viability [150]. There are several methods available to measure the number of cells that are in the process of dying (apoptosis). Apoptosis and preapoptosis evaluation methods, which are available for several models, can be used as early markers for cell viability through the use of specific fluorescent dyes (e.g., annexin V binding, apostain, tetramethylrhodamine, ethylester perchlorate-TMRE or DAPI labelling) [139,197]. Cell viability is probably the best described immune parameter in most species (Table 2); therefore, this parameter is one of most relevant to assess in cross-species comparison. In addition to measuring the overall immune cell viability, quantifying changes in the ratios of different subpopulations of immune cells (e.g., total hemocyte counts-THC) can often give a more detailed view of the impact of NPs on immune cell viability [141,179,199,200]. In human cells, fluorescence-activated cell sorting (FACS) is a common method in which fluorescent antibody-tags can be used to determine a large range of parameters but also to discriminate sub-cell populations [153]. 

The subcellular effects of NP exposure can be identified via assessment of the integrity of organelles, membranes, and other cellular compartments. Lysosomal functional integrity is an evolutionarily conserved marker of stress (including NPs) and of an individuals’ health status, and is commonly evaluated by measuring neural red retention or uptake [185,189,248]. Other approaches that can be used to assess the effects of NPs on organelles include methods measuring trans-golgi apparatus integrity and internal membrane polarization [178,179,200].

Another crucial step in the characterization of NP–immune system interactions is assessing whether immune cells are able to internalize NPs. The internalization of NPs has been observed for different types of NPs across the selected models and was recently reviewed in [36]. There are several techniques available to detect the internalization of NPs. These include transmission electron microscopy (TEM) which can image internalized particles and scanning electron microscopy (SEM) which helps to visualize membrane-bound particles and can give a direct image of the particles and cells following contact. They can also provide details on how the interaction occurs as well as the state of the NPs (e.g., agglomeration, aggregation, precipitation). These techniques when coupled to an EDX system (energy dispersive X-ray) can be used to perform chemical characterization of the NPs’ surfaces. TEM and SEM are descriptive techniques that can provide valuable information but makes quantification difficult between models. Some research has reported the use of fluorescently labelled particles to help to measure particle uptake, although the use of such labelled particles requires additional controls to rule out any effects linked to the leakage of fluorescent dyes [249]. In general, NP internalization in human cells has been well reported but for invertebrate, similar methods often require adjustments to be made (as for example, the salt or osmotic concentrations during fixation) [90,141,153,205,206,209]. For plants, TEM can be used to verify the entry of NPs into the cells [203]. In addition, these kinds of techniques can reveal the change in cell morphologies and subcellular structures (e.g., vacuoles, phagosomes, endosomes) upon NP exposure and give hints regarding the general activation or damages that the cell has undergone [203,207]. 

#### 3.4.2. Phagocytic Activity

While an immune response towards NPs could be part of normal immune functioning, overstimulation of the immune system resulting in damage or suppression leading to a compromised immune functioning may pose a threat to the organism. Such suppression of immune functioning caused by NPs could be studied via the assessment of the immune cells capacity to phagocytose and the consequent changes on index and rates (Figure 7, second point, Table 2). In earthworms, mussels, and sea urchins, phagocytosis can be evaluated by using fluorescence beads or yeast (using neutral red stained zymosan) [139,145,150,181,216,250].

#### 3.4.3. Cytotoxic Factors

Upon contact with NPs, cells can be activated and produce cytotoxic factors inside the cells in order to help to remove internalized foreign particles (Figure 7, third point). Among them, the oxidative burst, which involves the production of several radicals from oxygen (ROS) and nitrogen (RNS) derivatives. To quantify ROS, several methods, including the use of fluorescent probes (e.g., DCF or calcein), UV-vis spectroscopy (e.g., cytochrome C reduction) or histochemical staining, can be used and many of which have been adjusted for use across the model organisms (Table 2). Moreover, lipid peroxidation can be measured as a proxy for the damages caused by oxidative stress to the membranes, even if it is more frequently analyzed in tissues than in individual cells [138,139]. As for the quantification of RNS and more commonly nitric oxide (NO), in isopods, mussels, and earthworms, NO levels in the hemolymph can be measured spectrophotometrically from hemolymph samples using Griess reagent [141,234,250].

Lysozyme is an evolutionary conserved enzyme that catalyzes the hydrolysis of peptidoglycan and plays a role in the innate immunity of many organisms including earthworms, mussels, sea urchins, and plants [67,234,251,252]. The quantification of the release of lysozyme into the extracellular medium is based on the lysis activity of *Micrococcus lysodeikticus* which can be determined spectrophotometrically. Fluorescent probes can be also used to monitor the evolution of the lysosomal compartment and acidification in the cell upon exposure to NPs [200].

#### 3.4.4. Humoral Factors

Humoral immune responses play a crucial role in immunity by facilitating communication between immune cells and directing the extracellular destruction of foreign objects. Upon activation of the immune cells, some factors can be released into the extracellular medium (Figure 7, fourth point, Table 2). In mammals, cytokine production is a key driver of cellular immune responses [238]. Analyzing the extra- and intracellular concentration of cytokines secreted by (human) immune cells is a well-established method to test the effects and safety of NPs [253]. Many techniques have been developed to detect single or multiple cytokines and factors secreted by cells. These include classic methods such as western blot, ELISA, and bio-chemiluminescence assays, and many commercial possibilities for multiplex assays including legendplex or Ella multiplex technology [153,209,254]. Interestingly, in sea urchins, cytokine IL-6 can be detected in immune cells and secretome using western blot analysis after exposure to NPs [151]. 

Lysozyme and radicals can also be released by immune cells in the extracellular medium, to directly destruct foreign particles in close proximity to the cell. In addition, there are species specific released factors such as the hemolytic protein lysesin found in the coelomic fluid of earthworms. 

Lastly, an important immunological parameter often analyzed in invertebrate models is the measurement of phenoloxidase (PO) activity. This enzyme, produced via the pro-PO cascade, is involved in the production of melanin [43,141]. Upon detecting a melanized pathogen or object, immune cells quickly encapsulate the material resulting in the elimination of the threat. PO activity can be assessed by monitoring the formation of a reddish-brown pigments in the hemolymph from an individual organism using spectrophotometry [44,173,240]. In bivalves, the presence of PO has been reported but its basal levels, the variation across species and especially its response to NP exposure remain poorly understood [83,255]. In the sea urchin *Strongylocentrotus nudus* coelomocytes, three proteins with PO-like activities have been identified using electrophoretic methods [241].

#### 3.4.5. Molecular Response

Increasingly, humoral responses can be measured through genetic or omics approaches (e.g., quantitative PCR or full-transcriptome sequencing) (Table 2). A main advantage of using these approaches over biochemical ones is their high-throughput potential and increased specificity. Furthermore, genetic or omics approaches allow for the assessment of entire immunological pathways instead of focusing on specific biochemical endpoints. However, major limitations of these methods are that they require species specific primers and the availability of transcriptomes, which are currently lacking for many invertebrate species. Moreover, gene expression is highly regulated and time-dependent, so careful consideration must be given to the experimental model in terms of stimulation/exposure time, and cell collection technique. 

Genes involved in different immune-related functions, such as oxidative stress response, humoral factors (e.g., AMPs), and receptor proteins, are available for some organisms and the main immune related genes known to be activated upon NP exposure are reported in Table 2. 

A whole genome transcript is under development and will be available for *P. scaber*. Using this and genes previously annotated in other more commonly used crustacean species, primers specific for *P. scaber* immune-related genes can be designed (Hernadi, Mayall personal communication). Moreover, gene expression offers alternative possibilities to study several proteins involved in the immune response that biochemical tests that evaluate their activity or functionality are not feasible, such as the effects of NPs on AMP modulation. In plants, microarray-based studies are good tools to monitor the expression of candidate genes involved in the plant defense responses after interaction with NPs [204]. Additionally, an important future issue for environmental molecular biology is to establish whether an up- or downregulation of a certain gene correlates to a modification in the levels of related proteins [256]. The study of transcriptomic changes in cells, tissues, or full invertebrate organisms after exposure to NPs is now emerging but is still in its early phase. Transcriptome analysis can highlight pathways being activated but it should also be accompanied by the study of functional parameters for a fuller, deeper understanding [228,257,258]. In addition, changes in protein repertoires (proteomics approach) have shown interesting outcomes; however, studying of the combined immune response to NP exposure remains in its infancy [259,260,261].

## 4. Proposal for Future Cross-Species Evaluations and Conclusions

**Figure 8:**
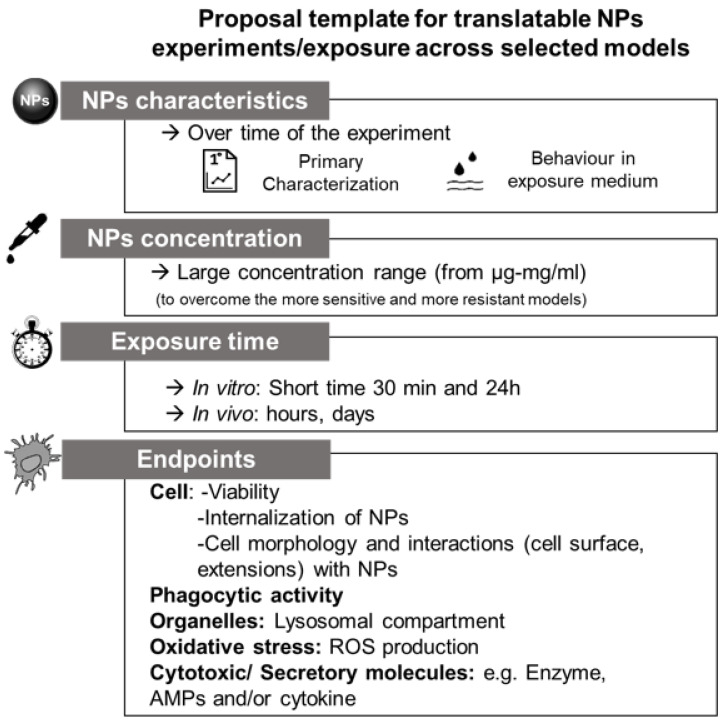
Proposal template for translatable NP experiments across the models of interest (plants, terrestrial and marine invertebrates, and human cells).

During the PANDORA project [35], several studies were conducted on the innate immune response of different models exposed to a large range of different NPs. Based on the outcomes of these studies, several conclusions can be made which may help to guide future (comparative) studies on nanoparticle–immune system interactions (Figure 8).

Because chemical conditions of mediums strongly affect the form and state of NPs and thereby the behavior of NPs, it is crucial to characterize the physico-chemical properties of NPs in the exposure medium as well as in their pristine form (e.g., after production). As the behavior and the interaction of NPs with immune systems are also time-dependent, experimental design will need to critically consider exposure duration as well. In vitro models can be considered as the prime focus for studying nanoparticle–immune system interactions. However, in vitro models are not available for all immune model species (e.g., isopods, plants), limiting comparative studies based on in vitro testing. In vivo experiments are crucial to study nanoparticle–immune system interactions under more realistic conditions. Studies using invertebrates, which are well-established and can be conducted on a routine basis, may serve as good alternatives to in vivo mammalian testing models such as mice or rats. 

Here, exposure route and exposure concentration will need to be critically considered as these factors are likely to significantly affect immune cells and their interaction with the NPs. 

In this review, we provided an overview of the methods used to characterize nanoparticle immune responses in various organisms across the tree of life (Table 2). Among cellular parameters, it appears that methods to assess cell viability (including assessments of subpopulations) and NPs internalization by immune cells are well described in most organisms. Phagocytic activity is a crucial parameter to be evaluated for immune cells, however, some models lack the methods to study this parameter (e.g., isopods) or do not rely on this type of response (e.g., plants). Moreover, as methods for microscopy are universally available, measurements of the change in morphology and external interactions of NPs can be studied in most immune models. 

Due to a lack of general knowledge on the composition and functioning of humoral immunity in most organisms other than mammal/human models, it remains difficult to identify the most relevant humoral parameter for cross-species evaluations. The exception being oxidative (and nitrosative) stress, for which methods are well described in most species.

Further work is needed to identify the interorganism comparability of otherwise species-specific markers, especially in invertebrates. In order to fully characterize NP-immune responses across species from the tree of life, there is a need for the identification of markers indicative for both pro- and anti-inflammatory responses, as are currently already available for human models (e.g., [253]). The development of such markers will require fundamental research on the innate immune systems of organisms other than human models. Thorough investigations in species from across the tree of life will help to understand how NPs interact with the innate immune system under different conditions and environments which may guide the future development of NPs that are immunologically safer-by-design. 

## Figures and Tables

**Table 1:** Summary of the main defense mechanisms involved in innate immunity at different levels of the models discussed.

Name	Innate Immune Cell Types	Whole Organism Level Defense	Cellular Response	Humoral/Extracellular Factors	Recognition & Activation
Plant *Arabidopsis thaliana*	All cells	Cell wall Waxy epidermal cuticle	MAMP-triggered immunity Effector triggered Immunity Hypersensitive response	ROS production Hormones (ethylene, JA, SA) Antimicrobial secreted peptides	PRRs: RLKs RLPs NLRs
Earthworm *Eisenia fetida*	Amoebocytes (granular and hyaline) Eleocytes	Skin Mucus Expulsion by dorsal pore Autotomy	Phagocytosis Agglutination-encapsulation ProPO cascade → melanization	AMPs (lumbricin I) Bacteriolytic enzyme (lysozyme) Hemolytic, proteolytic and cytotoxic proteins (fetidin and lysenins) ROS production	PRRs: CCF (lectinlike domain) TLR LBP/BPI
Terrestrial isopod *Porcelio scaber*	Hemocyte Granular and hyaline	Cuticle	Phagocytosis Encapsulation ProPO cascade → melanization	AMPs ROS/NO production	PRRs: TLR
Marine mussel *Mytilus galloprovincialis*	Hemocyte Granular and hyaline	Shell barrier Mucus layer Pseudo- feces	Phagocytosis Encapsulation ProPO	AMPs (mytilin, myticin, mytimicin), Defensins Complement system (C1qDC) Bacteriolytic enzyme-Lysozyme ROS /NO production	PRRs: lectins PGRPS TLR C1qDC FRED
Sea urchin *Paracentrotus lividus*	Macrophage-like phagocytes, amoebocytes (colorless, red); vibratile cells	Test Gut barrier Faeces	Phagocytosis Encapsulation	ROS production, AMPs (strongylocins, centrocins, paracentrin 1), lysozyme	PRRs: TLRs NLRs SRCR domain-containing proteins
Human	Monocytes Macrophages DCs Granulocytes ^1^	Epithelial and mucosal tissue	Phagocytosis Inflammation Granulocyte recruitment Antigen presentation	Complement antibodies, AMPs NETs, ROS/NO	PRRs:TLRs, NLRs, Scavenger Receptors, RLRs, CLRs,

^1^ Other innate cell types exist that are not discussed, including natural killer cells and innate lymphoid cells. Refer to the main text for the meaning of the abbreviations.

**Table 2:** Overview of studies demonstrating the use of cellular and humoral parameters to characterize the immune responses of organisms.

	Plants	Earthworms	Isopods	Mussels	Sea Urchins	Human
*1. Whole cell*						
**Cell viability**	✔	✔	✔	✔	✔	✔
LDH or ATP release	[180]	[181]				[153,182]
Fluorescent probes (FDA or PI)	[183,184]	[138,139]		[185,186]	[178]	[187]
Metabolic activity (MTT or CTB)	[188]	[181]		[189]		[153]
Blue tryptan	[190]	[191]	[173]	[192]	[193]	[194]
(Pre)-apoptosis (Annexin-V, DAPI, PI)	[195,196]	[138,139]	[141]	[197]	[121]	[198]
Cell subpopulation or polarization		[139,199]	[141,173]	[200,201]	[179]	[153,202]
**NP internalization**	✔	✔	✔	✔	✔	✔
TEM/SEM	[203,204]	[181,205]	[141]	[189,206,207,208]	[90,151,178]	[153,209]
**Organelles**	✔	✔	✖	✔	✔	✔
Neural red uptake/ release	[210,211]	[212]		[185,189]	[150,179]	[213]
Lysosome acidification	[214,215]			[200]	[179]	
Other organelles integrity (Trans-Golgi apparatus, Mitochondria)				[216]	[178,179]	
*2. Phagocytic activity*						
**Phagocytosis**	✖	✔	✖	✔	✔	✔
Phagocytic activity (index, rate)		[139,181]		[174,216]	[150,217]	[218]
*3. Cytotoxic factors*						
**Oxygen and nitrogen radicals**	✔	✔	✔	✔	✔	✔
ROS production	[219,220]	[139,205]		[146,208,221]	[151,222]	[223,224]
Lipid peroxidase activity	[220,225]	[138,139]		[226]		[227]
RNS (including NO) production	[228,229]	[230]	[173]	[185]		[231]
**Hydrolytic enzymes**	✔	✔	✖	✔	✔	✔
Lysozyme	[232]	[233]		[234]	[235,236]	[237]
Other species specific enzymes		lysenin [119]				
*4. Humoral factors*						
**Cytokines**	✖	✖	✖	✖	✔	✔
IL, TNF, IF secretion					[151]	[94,238,239]
**Melanization**	✖	✔	✔	✔	✔	✖
Phenoloxidase activation		[230,240]	[44]	[83]	[241]	
*5. Gene expression*						
**Oxidative stress genes**	✔	✔	✖	✔	✔	✖
Antioxidant defense and detoxification genes (e.g., CAT, SOD)	[242]	[140,195]		[176,200]	[243]	
**Circulating protein genes**	✖	✔	✖	✔	✖	✔
Signal transduction protein, enzymes, AMPs (general and species-specific)		Lysenin/Fetidin [141,170,192] CCF [181,244]		mytilin, myticin, EPp [176,200]		[231]
**Receptor protein genes**	✔	✔	✖	✔	✔	✔
TLR	[245]	[244]		[177]	[151,179]	[246]
LBP/BPI (LPS-binding protein/bacterial permeability-increasing protein)	[247]	[64]			[243]	
